# The Complete Mitochondrial Genome of *Bactrocera carambolae* (Diptera: Tephritidae): Genome Description and Phylogenetic Implications

**DOI:** 10.3390/insects10120429

**Published:** 2019-11-28

**Authors:** Elena Drosopoulou, Alexandros Syllas, Panagiota Goutakoli, Georgios-Alkis Zisiadis, Theodora Konstantinou, Dimitra Pangea, George Sentis, Alies van Sauers-Muller, Suk-Ling Wee, Antonios A. Augustinos, Antigone Zacharopoulou, Kostas Bourtzis

**Affiliations:** 1 Department of Genetics, Development and Molecular Biology, School of Biology, Faculty of Sciences, Aristotle University of Thessaloniki, 54124 Thessaloniki, Greece; aksyllas@bio.auth.gr (A.S.); giwta_gout@windowslive.com (P.G.); alkiszisiadis@gmail.com (G.-A.Z.); theodoki@bio.auth.gr (T.K.); mitsajohn@gmail.com (D.P.); sentigeo@yahoo.gr (G.S.); 2 Consultant, retired from Ministry of Agriculture, Animal Husbandry and Fisheries, Carambola Fruit Fly Project, Damboentong 282, Tijgerkreek, Saramacca, Suriname; aliesmuller@yahoo.com; 3 Center for Insect Systematics, Faculty of Science and Technology, Universiti Kebangsaan Malaysia, Bangi 43600, Selangor, Malaysia; slwee@ukm.edu.my; 4 Insect Pest Control Laboratory, Joint FAO/IAEA Division of Nuclear Techniques in Food and Agriculture, Seibersdorf, A-1400 Vienna, Austria; antoniosaugustinos@gmail.com (A.A.A.); K.Bourtzis@iaea.org (K.B.); 5 Biology Department, University of Patras, 26504 Patras, Greece; zacharop@upatras.gr

**Keywords:** *Bactrocera dorsalis* species complex, Carambola fruit fly, mitogenome, nucleotide polymorphisms, species delimitation, sterile insect technique

## Abstract

*Bactrocera carambolae* is one of the approximately 100 sibling species of the *Bactrocera dorsalis* complex and considered to be very closely related to *B. dorsalis*. Due to their high morphological similarity and overlapping distribution, as well as to their economic impact and quarantine status, the development of reliable markers for species delimitation between the two taxa is of great importance. Here we present the complete mitochondrial genome of *B. carambolae* sourced from its native range in Malaysia and its invaded territory in Suriname. The mitogenome of *B. carambolae* presents the typical organization of an insect mitochondrion. Comparisons of the analyzed *B. carambolae* sequences to all available complete mitochondrial sequences of *B. dorsalis* revealed several species-specific polymorphic sites. Phylogenetic analysis based on *Bactrocera* mitogenomes supports that *B. carambolae* is a differentiated taxon though closely related to *B. dorsalis*. The present complete mitochondrial sequences of *B. carambolae* could be used, in the frame of Integrative Taxonomy, for species discrimination and resolution of the phylogenetic relationships within this taxonomically challenging complex, which would facilitate the application of species-specific population suppression strategies, such as the sterile insect technique.

## 1. Introduction 

The *Bactrocera dorsalis* species complex consists of approximately 100 morphologically similar taxa distributed mainly in South-East Asia and Australasia [1]. Although most members within the complex present no economic interest, a small number of them are serious pests infesting many commercial fruits. Among them are the Oriental fruit fly, *B. dorsalis* (including the species formerly known as *B. philippinensis*, *B. papayae* and *B. invadens*), and the Carambola fruit fly*, B. carambolae,* both of which are highly destructive and invasive [2]. Hence, the clarification of their phylogenetic relationships and the development of robust species discriminating tools for the above taxa presents not only scientific, but also great economic interest as the outcome potentially affects international trade regulations and quarantine policies. In the frame of Area-Wide Integrated Pest Management (AW-IPM), availability of population-specific and species-specific markers is also critical. As examples, such markers can support the quick identification of the origin of new invasions or expansions of pests as well as the development and application of species-specific pest control methods that include mass rearing and release of laboratory insects to suppress local populations, such as the sterile insect technique (SIT), since they allow both assessing the suitability of different strains for local applications and identifying released males [3,4].

Species delimitation among the members of the *B. dorsalis* complex has been a long-standing issue because of their overlapping geographical distributions [1,5], overlapping host range [6], the lack of prominent discriminating morphological characteristics and the significant intraspecific morphological variations [7]. Recently, three taxa have been synonymized as one biological species: *B. philippinensis* (hereafter *B. ‘*syn. *philippinensis’*) was first synonymized by Drew and Romig [1] as *B. papayae* (hereafter *B. ‘*syn. *papayae’*) while Schutze et al. [8] subsequently synonymized both *B. ‘*syn. *papayae’* and *B. invadens* (hereafter *B. ‘*syn. *invadens’*) as *B. dorsalis.* The latter taxonomic revision was supported by multidisciplinary evidence from morphological, morphometric, molecular/genetic, cytogenetic, behavioral/sexual compatibility and chemo-ecological studies [8,9,10,11,12,13,14,15,16,17,18,19,20,21]. However, the synonymization by Schutze et al. [8] was criticized [22] and debated [23]. The species status of *B. carambolae* in relation to *B. dorsalis* has also been explored by many researchers using multiple approaches. Data on morphology/morphometrics [1,24], certain genetic markers [12,14,21,25,26,27,28,29], mating behavior [11] and chemoecology [20,30,31,32] supported the identity of *B. carambolae* as a separate biological species and provided some diagnostic features for species discrimination. On the other hand, identification of morphological hybrids [33] and data from nuclear protein coding genes [14] and microsatellite analysis [34] suggest naturally occurring hybridization and gene flow between the two taxa.

Mitochondrial DNA (mtDNA) is a very popular molecular marker for evolutionary, phylogenetic and population genetic studies and is informative for analyses at several taxonomic levels [35]. Partial mitochondrial sequences have been extensively used for exploring relationships among species of the *Bactrocera* genus; however, they had their limitations, for instance in the discrimination among closely related members of the *B. dorsalis* complex [9,10,12,14,15,25,27,36,37,38,39,40,41]. On the other hand, complete mitochondrial genome sequences, which are accumulating rapidly in databases nowadays, have proven to be a valuable alternative approach for phylogeny reconstruction and molecular systematics in several insect groups [42,43,44,45,46,47,48,49,50], including Tephritidae [51,52,53,54,55,56,57,58,59,60,61,62,63,64]. Especially, when the discrimination of closely related species is attempted, the comparative analysis of complete mitogenomes can help to select the most informative mitochondrial markers/sequences for specific issues [53]. However, it is becoming more evident that factors related to mtDNA inheritance, such as bottlenecks, introgression, heteroplasmy and sweeps by reproductive symbionts, restrict the usefulness of mtDNA as a standalone marker for species delimitation [65,66,67]. Therefore, the combined use of both mitochondrial and nuclear genetic markers together with information from different disciplines is expected to provide a more accurate and indisputable species resolution under the umbrella of Integrative Taxonomy [68].

In the current study, the complete mitochondrial genome sequences of three well-characterized *B. carambolae* specimens originating from the native as well as from the invaded territory of the species were generated and described in detail. The new *Β. carambolae* mitogenomes together with the one that was already available in the databases were compared against three complete mitochondrial sequences of *B. dorsalis* generated in the present study and all available ones from the databases, including *B*. *‘*syn. *philippinensis’*, *B*. *‘*syn. *papayae*’ and *B*. *‘*syn. *invadens’*, attempting to identify potential species-specific polymorphic sites throughout the mitogenome. Furthermore, a phylogenetic analysis within *Bactrocera* was performed focusing on the placing of the *B. carambolae* complete mitogenomes in comparison to *B. dorsalis*.

## 2. Materials and Methods

### 2.1. Insects 

The *B. carambolae* specimens used in this study originated from Malaysia and Suriname. The Malaysian strains were collected from infested wax apples (*Syzygium* spp.) from the forest fringe in Raub, Pahang state, Malaysia. The emerged adults from the infested fruits were subjected to morphological identification based on three key morphological characteristics: (a) the presence of a recurved pattern on the wing costal band beyond apex R^4+5^, (b) the presence of a fore femoral dark spot and (c) the presence of bar-shaped bands at terga III–V [3]. Close morphological identification confirmed all flies emerged from this source was *B. carambolae*. Flies were laboratory reared for 2–3 generations on carambola (*Averrhoa carambola*) fruits (27 ± 2 °C, 85% ± 5% RH, 12 h L: 12 h D) in order to confirm the species status of the offspring and to raise a sufficient number of flies. The sample from Suriname came from a laboratory colony initiated by insects collected from carambola fruits from the districts of Paramaribo and Saramacca, Suriname, and reared on carambola fruits for 21 generations (24 °C, 85% RH, 12 h L: 12 h D) in the Carambola fruit fly unit, Department of Agricultural Research, Ministry of Agriculture, Animal Husbandry and Fisheries, Paramaribo. Pupae from the above strains were sent to the Insect Pest Control Laboratory (IPCL) of the Joint FAO/IAEA Division of Nuclear Techniques in Food and Agriculture (Seibersdorf, Austria) and adults emerging from these pupae were used in the present study. In addition, *B. dorsalis* specimens from laboratory colonies maintained on artificial diet (25 ± 2 °C, 60% ± 5% RH, 14 h L: 10 h D) at the IPCL were used. The above colonies represented three populations originating from Saraburi (Thailand), Philippines (*B. ‘*syn. *philippinensis’*) and Kenya (*B. ‘*syn. *invadens’*). Their status has been verified by taxonomists and the insect materials have been used in several research projects [11,17,18,19,20,69].

### 2.2. DNA Isolation, Amplification and Sequencing 

Total genomic DNA was extracted from single flies, using either the CTAB protocol [70] or the DNeasy Blood and Tissue kit (Qiagen, Hilden, Germany) following manufacturer’s instructions for total DNA purification from animal tissues. Negative controls were included in DNA extraction. DNA quality and quantity were measured using the NanoDrop 1000 Spectrophotometer (Thermo Fischer Scientific, Waltham, MA, USA).

Each mitogenome sequence was obtained from a single specimen by standard PCR amplifications using primers that were designed based on the mitochondrial sequence of *Bactrocera dorsalis* (accession no NC_008748; Supplementary Table S1). Twenty-seven pairs of primers targeting overlapping fragments were designed by the Oligoexplorer and Oligoanalyzer programs (Supplementary Table S2). Approximately 30 ng of template DNA was used in each reaction of 25 μL (1× PCR buffer, 1.5 mM MgCl_2_, 0.2 mM of each dNTP, 0.5 μM of the appropriate primers and 1 U *Taq* polymerase). The BIOTAQ (BIOLINE, UK) or the One Taq (NEB, Ipswich, MA, USA) DNA polymerases were used. Amplification was performed in a SensoQuest thermocycler by the following program: initial denaturation at 94 °C for 3 min, 40 cycles of 45 section denaturation at 94 °C, 30 section primer annealing at 46–59 °C and 1 min DNA chain extension at 72 °C, and final extension at 72 for 7 min. PCR products were purified by the Nucleospin Gel and PCR Clean up kit (Macherey Nagel, Düren, Germany) or by Exonuclease I and Shrimp Alkaline Phosphatase (NEB, USA).

Sequencing reactions were performed by Macrogen Europe (Amsterdam, The Netherlands) or Eurofins Genomics (Ebersberg, Germany). Each fragment was sequenced in both directions and the sequences obtained by the forward and the reverse reactions were merged using EMBOSS Merger [71] after careful manual inspection. In cases of inconsistencies reactions were repeated. The mitogenome sequences were assembled using EMBOSS Merger [71] and submitted to GenBank (accession nos.: KT343905, MG916998, MN104217-20, Supplementary Table S1).

### 2.3. Sequence Analysis 

Sequence annotation was manually performed by comparison to the *B. dorsalis* mitogenome sequence (accession no NC_008748; Supplementary Table S1). The secondary structure and the presence of specific anticodons of the 22 *tRNA*s were checked by tRNAscan-SE [72] (http://lowelab.ucsc.edu/tRNAscan-SE/) and MITOS [73] (http://mitos.bioinf.uni-leipzig.de/index.py). Repeats in the control region were found by the “Tandem Repeat Finder” program [74] (http://tandem.bu.edu/trf/trf.html). Multiple sequence alignments for genome annotation as well as for identification of polymorphic sites were performed by ClustalOmega (www.ebi.ac.uk) using default parameters.

### 2.4. Phylogenetic Analysis 

Phylogenetic analysis based on concatenated *COI* and *ND4* partial gene sequences was performed using *B. dorsalis* and *B. carambolae* sequences from different locations previously analyzed by Boykin et al. [27] (Supplementary Table S3) together with the corresponding gene fragments from the complete sequences generated in the present study (Supplementary Table S1) (dataset 1). Phylogenetic analysis based on alignments of complete mtDNA sequences was performed using all *Bactrocera* complete mitogenomes available (Supplementary Table S1) (dataset 2). Multiple sequence alignments were constructed by ClustalW using default parameters. Phylogenetic trees were inferred by the Maximum Likelihood (ML) method based on the Hasegawa–Kishino–Yano model (dataset 1) or the General Time Reversible (GTR) (dataset 2) model with 1000 bootstrap replicates. All the above analyses, alignments, model selection and phylogeny reconstruction, were performed in MEGA 7.0 [75,76,77]. Dataset 2 was also analyzed by maximum likelihood (ML) inference using IQ-TREE 1.4.2 [78] and, in particular, the IQ-TREE web server (http://iqtree.cibiv.univie.ac.at) [79]. The best-fit substitution model was determined by IQ-TREE (“Auto” option in the field *Substitution model*) including FreeRate heterogeneity in the model selection process (“Yes [+R]” option in the field *FreeRate heterogeneity*). To assess nodal support, 1000 ultrafast (UFBoot) [80] bootstrap replicates were performed.

## 3. Results and Discussion

The mitogenomes of three *B. carambolae* specimens, two from Malaysia (M5 and M8) and one from Suriname (S2) were analyzed. In order to further substantiate the species characterization of the specimens, we performed a phylogenetic analysis based on *COI* + *ND4* partial mitochondrial sequences from the *B. dorsalis* complex previously used in several studies [10,12,27] together with the ones generated in the present study (Supplementary Tables S1 and S3). The above analysis clustered the sequences of our specimens together with the other *B. carambolae* and separately from all *B. dorsalis* sequences (Supplementary Figure S1), which, although in a context of low nodal support, supports their identification as true representatives of *B. carambolae*.

**Figure 1:**
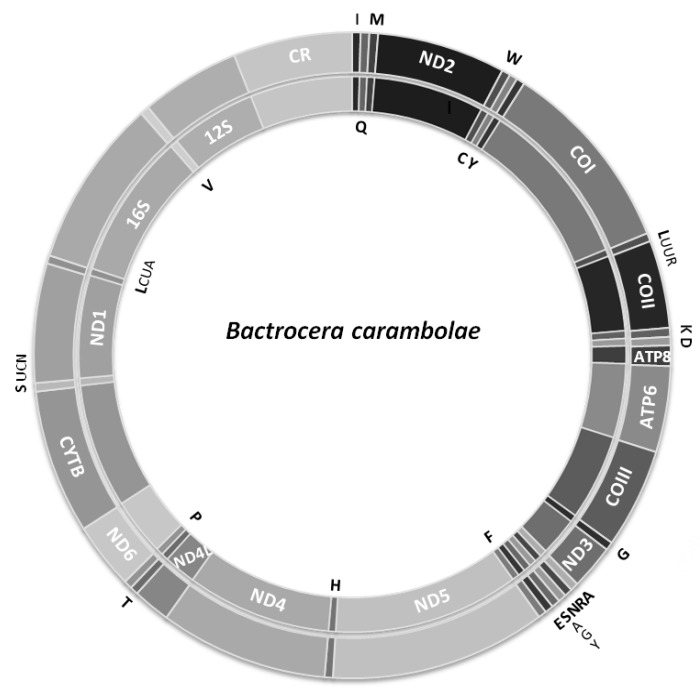
The *Bactrocera carambolae* mitochondrial genome. Genes shown at the outer circle are encoded by the H-strand whereas those at the inner circle are encoded by the L-strand. Abbreviations as in Table 1.

The mitogenomes of the M8 and S2 individuals were completely sequenced and found to be of 15,918 and 15,912 bp long, respectively. Frοm the M5mitogenome, 15,034 bp were successfully sequenced, while part from the non-coding region between the 12S rRNA and tRNAIle genes of about 900 bp in size was missing. Each mitogenome contains 13 protein-coding, two rRNA (12S and 16S rRNA) and 22 tRNA genes, and one major non-coding sequence, the control region (Figure 1; Table 1). All mitogenomes presented very high A + T content (72.64–72.75%, excluding control region) with gene arrangements identical to other *Bactrocera* mitogenomes [51,53,54,55,56,62,63,64,81,82,83].

### 3.1. Protein-Coding Genes

The majority of the protein-coding genes (PCGs) are encoded by the H strand and only *ND1*, *ND4*, *ND4L* and *ND5* are encoded by the L strand (Table 1). The initiation codons were identical to those reported for *B. dorsalis* PCGs [53], i.e., ATG for *COII*, *ATP6*, *COIII*, *ND4*, *ND4L* and *CYTB*; ATT for *ND2*, *ND3*, *ND5* and *ND6*; ATA for *ND1*; TCG for *COI* and GTG for *ATP8* (Table 1). The GTG initiation codon seems to be characteristic for the *ATP8* gene of the species of the *Bactrocera* subgenus [53,54,55,56,62,63,64,83,84]. Three of the PCGs possess an incomplete termination codon (Table 1); TA for *COI*, which is characteristic for all *Bactrocera* species analyzed so far and T for *ND1* and *ND5* similarly to the majority of tephritids [52,53,54,55,56,57,58,59,60,61,62,63,64,81,82,83,84,85,86,87,88]. Incomplete termination codons are common in animal mitochondrial DNA and are likely to be completed by post-transcriptional polyadenylation [89].

The overlaps of seven nucleotides between *ATP8* and *ATP6* and *ND4* and *ND4L* genes are the longest observed between protein-coding genes of *B. carambolae.* Overlaps restricted to one or two nucleotides can also be observed between *ATP6* and *COIII*, *ND3* and *tRNA^Ala^*, *ND6* and *CYTB* and *CYTB* and *tRNA^Ser^* (Table 1). Overlaps that are similar in size and position are common among tephritids [52,55,58,59,60,61,62,63,64].

### 3.2. RNA Genes

The *16S rRNAs* of *B. carambolae* M5 and S2 individuals consist of 1332 nucleotides (positions: 12,776–14,107 and 12,773–14,104, respectively), while that of the M8 appears to be one nucleotide shorter (positions: 12,773–14,103) (Table 1). Similarly, the *12S rRNA* genes are 790 nucleotides long for M5 and S2 and 789 for M8 (positions: 14,180–14,969, 14,177–14,966 and 14,176–14,964, respectively) (Table 1). In accordance with other insect mitogenomes, these genes are located in the L strand between the gene for *tRNA^Leu^* (CUA) and the control region, and are separated by the *tRNA^Val^* gene (Table 1). The 22 *tRNA* genes, predicted to fold into the expected cloverleaf secondary structures, are dispersed among the protein-coding and the *rRNA* genes; 14 of them lie on the H and 8 on the L strand of the mtDNA (Table 1). Their positions and sizes (63–72 nucleotides) follow the typical organization for insect mtDNA.

### 3.3. Non-Coding Regions

Similarly, to all tephritids, the mitogenome of *B. carambolae* contains only one long non-coding region, i.e., the control region (D-loop), located between the 12S *rRNA* and the *tRNA^Ile^* genes (Figure 1). Its length was found to be 949 and 948 nucleotides and its A+T content was found to be 87.14% and 87.87% for M8 and S2 individuals, respectively (Table 1).

A stretch of 22 thymidines resides at the 5′ end of the D-loop (near to the *tRNA^Ile^* gene), a feature that is common among tephritid and other insect mitogenomes, and is believed to play a role in the control of transcription and/or replication [54,59,60,81,90]. The 13 nucleotide long motifs TTTAATTTTTTAA and TTAATTTTATTAA were found to be tandemly repeated four times at the same position (D-loop position 212–262) of the M8 and S2 individuals, respectively. Tandem repeats have been identified in the control regions of *Bactrocera* as well as in other tephritid species [54,60,81].

The longest intergenic spacer (IGS) region in the analyzed *B. carambolae* mitogenomes was found between the *tRNA^Gln^* and *tRNA^Met^* genes with a size of 66 nucleotides for both M5 and M8 individuals and 67 nucleotides for S2 (Table 1). Although the position of the longest IGS seems to be conserved among several species of the *Bactrocera* subgenus [53,55,56,62,63], the sequence presents no significant similarity except within the *B. dorsalis* complex (the sequence identity between *B. carambolae* and *B. dorsalis* was about 97%). The second longest IGS is located between the *tRNA^Cys^* and *tRNA^Tyr^* genes and is 46 nucleotides long in all three *B. carambolae* specimens analyzed (Table 1). This IGS folded into secondary structures and its first 33 nucleotides could be found repeated in the D-loop region of *B. carambolae*, which is similar to other *Bactrocera* species as well as members of the *B. dorsalis* complex suggesting recombination events [53,81]. Yu et al. [53] reported an 11 bp insertion at the end of this spacer in a *B. carambolae* specimen and suggested that it could be used as a specific marker for species discrimination between *B. carambolae* and the other members of the complex. However, the above insertion was not observed in any of the three *B. carambolae* specimens analyzed. Furthermore, a short TA repeat making this IGS longer (53 compared to 46 bp) was also found in one of the *B. dorsalis* sequences generated in the present study (accession no KT343905). The above findings suggest that small insertions in the spacer lying between the *tRNA^Cys^* and *tRNA^Tyr^* genes are more likely to represent individual- or population- rather than species-specific polymorphisms.

### 3.4. Sequence Comparisons and Phylogenetic Analysis

The three *B. carambolae* mitogenomes analyzed here were compared to the complete mitochondrial sequences of *B. carambolae* (one) and *B. dorsalis* (six) found in GenBank and to the three additional sequences of the *B. dorsalis* complex generated in the present study (Supplementary Table S1). The identity scores obtained between the complete mitogenome sequences from *B. carambolae* and *B. dorsalis* ranged from 98.45% to 98.98% being imperceptibly lower than the ones observed among the *B. dorsalis* sequences (98.88–99.49%). The identity scores were extremely high even for the D-loop region, which is considered the most variable region of the mitogenome (95.68–98.52% between *B. carambolae* and *B. dorsalis*; 97.99–99.16% among *B. dorsalis*).

However, alignment of the above sequences revealed a small number (12) of positions that consistently differed between the *B. carambolae* and the *B. dorsalis* sequences (Table 2). Almost all of the above polymorphisms were found within the PCG sequences and could be potential markers for discriminating the two very closely related taxa analyzed. Nonetheless, additional data at population level is required to assess whether these polymorphisms are fixed and species-specific.

**Figure 2:**
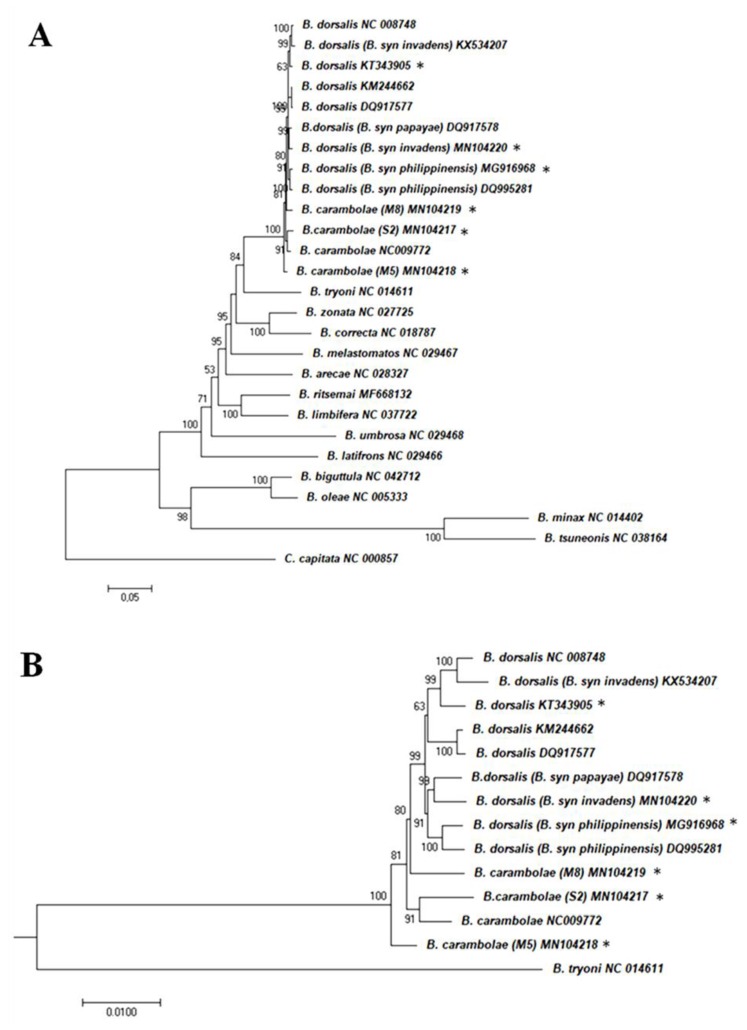
Molecular phylogenetic analysis by Maximum Likelihood method. (**A**) Tree based on 26 *Bactrocera* complete mitochondrial genome sequences; (**B**) part of the tree depicted in (**A**) presenting only the clade of the *B. dorsalis* complex sequences. *Ceratitis capitata* was used as outgroup to root the tree. The evolutionary history was inferred by using the Maximum Likelihood method based on the General Time Reversible model. The percentage of trees in which the associated taxa clustered together is shown next to the branches; only the ones higher than 50 are presented. The tree is drawn to scale, with branch lengths measured in the number of substitutions per site (scale bar = 0.05 (**A**) or 0.01 (**B**) substitutions per site). Asterisks indicate the sequences analyzed in the present study. Sequences’ accession numbers in Supplementary Table S1.

The ML phylogenetic analysis with the complete *Bactrocera* mitochondrial sequences (six generated in the present study and 20 from GenBank) (Supplementary Table S1) conducted with MEGA software (Figure 2) resulted in almost identical tree topology to the one inferred by the IQ-Tree ML algorithm (data not shown). Topologies were very similar to other recent analyses also using data of complete mitogenomes to explore phylogenetic relationships within the *Bactrocera* genus [54,58,63,82,83,91,92] and confirmed the very close relationship of the *B. dorsalis* complex members. Within the complex (Figure 2B), the *B. dorsalis* sequences formed a highly supported clade, while all *B. carambolae* sequences, though not forming a single clade, were placed outside the *B*. *dorsalis* clade. The above results suggest the differentiation of the *B. carambolae* mitosequence and could provide some support to the retention of *B. carambolae* as a separate taxon from *B. dorsalis* [8]. However, additional data and analyses would be required to clarify the issue of species limits between the above two taxa.

In summary, the complete mitochondrial sequence of three *B. carambolae* specimens is presented. These are the first published *B. carambolae* mitogenomes described in detail, though not the first appearing in databases. The structure and the organization of the *B. carambolae* mitogenomes analyzed follow the typical pattern of an insect mitochondrion. The availability of several complete *B. carambolae* mitogenomes allowed, through sequence alignments against all available *B. dorsalis* mitogenomes, the identification of potentially species-specific nucleotide polymorphisms. Phylogenetic analyses within the *Bactrocera* genus supported the differentiation of *B. carambolae* in comparison to *B. dorsalis.* The future disposal of additional complete mitosequences from other members of the *B. dorsalis* complex could enable more extensive comparative analyses, to aim for a better resolution of their evolutionary relationships and for identification of the most informative polymorphic mitochondrial regions. Nevertheless, multidisciplinary approaches, combining mitochondrial and nuclear genetic information together with data on different aspects of species biology in the frame of Integrative Taxonomy, are considered necessary for reliable identification of species boundaries within this speciose complex of destructive pests.

## Figures and Tables

**Table 1:** Organization of the *Bactrocera carambolae* mitochondrial genome.

Gene/Element	Abbreviation	Strand	Start Position M5 M8 S2			Size (bp) M5/M8/S2	IGS (after) M5/M8/S2	Start Codon	Stop Codon
*tRNA^Ile^*	*I*	H	1	1	1	66	−3		
*tRNA^Gln^*	*Q*	L	64	64	64	69	66/66/67		
*tRNA^Met^*	*M*	H	199	199	200	69	0		
*NADH dehydrogenase subunit 2*	*ND2*	H	268	268	269	1023	10	ATT	TAA
*tRNA^Trp^*	*W*	H	1301	1301	1302	69	−8		
*tRNA^Cys^*	*C*	L	1362	1362	1363	63	46		
*tRNA^Tyr^*	*Y*	L	1471	1471	1472	67	−2		
*Cytochrome c oxidase subunit 1*	*COI*	H	1536	1536	1537	1535	0	TCG	TA *
*tRNA^Leu^ (* *UUR* *)*	*L_UUR_*	H	3071	3071	3072	66	4		
*Cytochrome c oxidase subunit 2*	*COII*	H	3141	3141	3142	690	4	ATG	TAA
*tRNA^Lys^*	*K*	H	3835	3835	3836	71	0/0/2		
*tRNA^Asp^*	*D*	H	3906	3906	3909	67	0		
*ATP synthase F0 subunit 8*	*ATP8*	H	3973	3973	3976	162	−7	GTG	TAA
*ATP synthase F0 subunit 6*	*ATP6*	H	4128	4128	4131	678	−1	ATG	TAA
*Cytochrome c oxidase subunit 3*	*COIII*	H	4805	4805	4808	789	9	ATG	TAA
*tRNA^Gly^*	*G*	H	5603	5603	5606	65	0		
*NADH dehydrogenase subunit 3*	*ND3*	H	5668	5668	5671	354	−2	ATT	TAG
*tRNA^Ala^*	*A*	H	6020	6020	6023	65	7		
* tRNA^Arg^*	*R*	H	6092	6092	6095	64	11		
* tRNA^Asn^*	*N*	H	6167	6167	6170	65	0		
*tRNA^Ser^(* *AGY* *)*	*S_AGY_*	H	6232	6232	6235	68	0		
* tRNA^Glu^*	*E*	H	6300	6300	6303	67	18		
* tRNA^Phe^*	*F*	L	6385	6385	6388	65	0		
*NADH dehydrogenase subunit 5*	*ND5*	L	6450	6450	6453	1720	15	ATT	T *
* tRNA^His^*	*H*	L	8185	8185	8188	66	0		
*NADH dehydrogenase subunit 4*	*ND4*	L	8251	8251	8254	1341	−7	ATG	TAG
*NADH dehydrogenase subunit 4L*	*ND4L*	L	9585	9585	9588	297	2	ATG	TAA
* tRNA^Thr^*	*T*	H	9884	9884	9887	65	0		
* tRNA^Pro^*	*P*	L	9949	9949	9952	66	2		
*NADH dehydrogenase subunit 6*	*ND6*	H	10,017	10,017	10,020	525	−1	ATT	TAA
*Cytochrome b*	*CYTB*	H	10,541	10,541	10,544	1137	−2	ATG	TAG
*tRNA^Ser^(UCN)*	*S_UCN_*	H	11,676	11,676	11,679	67	15		
*NADH dehydrogenase subunit 1*	*ND1*	L	11,758	11,758	11,761	940	10	ATA	T *
* tRNA^Leu^* *(CUA)*	*L_CUA_*	L	12,708	12,708	12,711	65	1		
* 16S rRNA *	*16S*	L	12,773	12,773	12,776	1332/1331/1332	0		
* tRNA^Val^*	*V*	L	14,105	14,104	14,108	72	0		
* 12S rRNA *	*12S*	L	14,177	14,176	14,180	790/789/790	0		
Control region	CR		14,967	14,965	14,970	− ^#^/948/949	0		

* TAA stop codon is completed by the addition of 3′A residues to mRNA. ^#^ Symbol “−” indicates missing information.

**Table 2:** The interspecies nucleotide polymorphisms observed in the complete mitochondrial sequences of *B. dorsalis* and *B. carambolae* used in the present study. Gene abbreviation as in Table 1 Position refers to nucleotide position within respective gene.

Gene	Position	Nucleotide in *B. dorsalis*	Nucleotide in *B. carambolae*
*ND2*	53	C	T
	467	G	A
	473	C	T
*COI*	425	C	T
	933	G	A
*ATP6*	120	C	T
	675	C	T
*COIII*	69	T	C
*ND5*	548	A	G
*ND4*	502	A	G
*CYTB*	462	C	T
*16S*	23	T	C

## Supplementary Materials

The following are available online at https://www.mdpi.com/2075-4450/10/12/429/s1, Figure S1. Molecular phylogenetic analysis by Maximum Likelihood method based on concatenated partial sequences of the *COI* and *ND4* genes of 38 *B. carambolae* and *B. dorsalis* specimens, Table S1. List of the mitogenome sequences used in the present study, Table S2. List of the primers used for the amplification of the mitogenomes of the *Bactrocera carambolae* and *Bactrocera dorsalis* specimens, Table S3. List of the GenBank accession numbers of the COI and ND4 partial sequences from *B. dorsalis* and *B. carambolae* used in the present study.
