# Supplementary material for: The Complete Mitochondrial Genome of Bactrocera carambolae (Diptera: Tephritidae): Genome Description and Phylogenetic Implications
Source: Insects. 2019 Nov 28;10(12):429. doi: 10.3390/insects10120429 (PMC6955806; doi:10.3390/insects10120429)
Supplement: Supplementary file 1 [file insects-10-00429-s001.zip › Drosopoulou et al._Supplementary information_revised.docx]

**Supplementary Information**

**Τhe complete mitochondrial genome of *Bactrocera carambolae* (Diptera: Tephritidae): genome description and phylogenetic implications**

**Elena Drosopoulou^1*^, Alexandros Syllas^1^, Panagiota Goutakoli^1^, Alkis-George Zisiadis^1^, Theodora Konstantinou^1^, Dimitra Pangea^1^, George Sentis^1^, Alies van Sauers-Muller^2^, Suk-Ling Wee^3^, Antonios A. Augustinos^4§^, Antigone Zacharopoulou^5^, Kostas Bourtzis^4^**

^1^Department of Genetics, Development and Molecular Biology, School of Biology, Faculty of Sciences, Aristotle University of Thessaloniki, Thessaloniki, Greece

^2^Consultant, retired from Ministry of Agriculture, Animal Husbandry and Fisheries, Carambola fruit fly project, Paramaribo, Suriname^.^

^3^Center for Insect Systematics, Faculty of Science and Technology, Universiti Kebangsaan Malaysia, 43600 Bangi, Selangor, Malaysia

^4^Insect Pest Control Laboratory, Joint FAO/IAEA Division of Nuclear Techniques in Food and Agriculture, Seibersdorf, Vienna, Austria

^5^Biology Department, University of Patras, Patras, Greece

^§^ Present address: Department of Plant Protection, Institute of Industrial and Forage Crops, Hellenic Agricultural Organization – DEMETER, Patras, Greece

* Corresponding author

Elena Drosopoulou

Department of Genetics, Development and Molecular Biology,

School of Biology, Faculty of Sciences, Aristotle University of Thessaloniki (AUTH),

GR-54124, Thessaloniki, Greece.

Tel: + 30 2310 998291, Fax: + 30 2310 998333

e-mail: [edrosopo@bio.auth.gr](mailto:edrosopo@bio.auth.gr)

**Supplementary Table S1.** List of the complete mitogenome sequences used in the present study. Species names, abbreviation used, GenBank accession numbers and references are given.

| Species | GenBank  Accession number | Reference |
| --- | --- | --- |
| *Bactrocera dorsalis* | NC_008748 | [53] |
| *Bactrocera dorsalis* | DQ917577 | *-* |
| *Bactrocera dorsalis* | KM244662 | [93] |
| *Bactrocera dorsalis (B. `syn. papayae’)* | DQ917578 | *-* |
| *Bactrocera dorsalis (B. `syn. philippinensis'*) | DQ995281 | *-* |
| *Bactrocera dorsalis (B. `syn. invadens’)* | KX534207 | [82] |
| *Bactrocera dorsalis* | KT343905 | Present study |
| *Bactrocera dorsalis (B. `syn. invadens’)* | MN104220 | Present study |
| *Bactrocera dorsalis (B. `syn. philippinensis'*) | MG916968 | Present study |
| *Bactrocera carambolae* | NC_009772 | *-* |
| *Bactrocera carambolae (M5)* | MN104218 | Present study |
| *Bactrocera carambolae (M8)* | MN104219 | Present study |
| *Bactrocera carambolae (S2)* | MN104217 | Present study |
| *Bactrocera correcta* | NC_018787 | [83] |
| *Bactrocera zonata* | NC_027725 | [54] |
| *Bactrocera arecae* | NC_028327 | [55] |
| *Bactrocera tryoni* | NC_014611 | [52] |
| *Bactrocera melastomatos* | NC_029467 | [56] |
| *Bactrocera latifrons* | NC_029466 | [56] |
| *Bactrocera umbrosa* | NC_029468 | [56] |
| *Bactrocera ritsemai* | NC_037723 | [62] |
| *Bactrocera limbifera* | NC_037722 | [63] |
| *Bactrocera oleae* | NC_005333 | [51] |
| *Bactrocera biguttula* | MK293875 | [92] |
| *Bactrocera minax* | NC_014402 | [81] |
| *Bactrocera tsuneonis* | NC_038164 | [64] |
| *Ceratitis capitata* | NC_000857 | [94] |

**Supplementary Table S2.** List of the primers used for the amplification of the mitogenomes of the *Bactrocera carambolae* and *Bactrocera dorsalis* specimens. Numbers in primer names indicate the exact primer position in the *B. dorsalis* mtDNA sequence (NC_008748). F: forward primer, R: reverse primer.

| **Primer name** | **Sequence 5′-3′** | **Amplicon size** (bp) |
| --- | --- | --- |
| **Bd 317 F**  **Bd 846 R** | GAACCCTAATCACTGTATCCTC | 530 |
|  | TTCACTTGCTTGTATGGCTGC |  |
| **Bd 757 F**  **Bd 1623 R** | GACTTAATCAAACTTCTCTGCG | 867 |
|  | GATGTTCCTACTATTCCTGCTC |  |
| **Bd 1507 F**  **Bd 2293 R** | GTCTATCGCCTAAACTTCAGC | 787 |
|  | GGAAATTATCCCGAATCCTGG |  |
| **Bd 2160 F**  **Bd 3056 R** | CAGACCGAAACTTAAATACTTCC | 897 |
|  | GAGGTAGTTCTGAATAACTGTG |  |
| **Bd 2850 F**  **Bd 3656 R** | CAGACTATCCAGATGCTTACAC | 807 |
|  | CTACCTTTACACCTAAGGCTG |  |
| **Bd 3518 F**  **Bd 4251 R** | GAATTAGCAACAGACGGATTCC | 734 |
|  | CGTGAGGGTATTAATCAGTAGG |  |
| **Bd 3968 F**  **Bd 4650 R** | TTTGGTGCCTCAAATAGCCC | 683 |
|  | AGAGTAAGTAATAAATGTCCTGC |  |
| **Bd 4508 F**  **Bd 5137 R** | GCTCACCTAGTACCTCAAGGAACC | 630 |
|  | CAATGGCTGGAGATAAACTTCTGTGG |  |
| **Bd 5014 F**  **Bd 5814 R** | CTACACACCTTACCTGTAACATTAGG | 801 |
|  | GTGAAGAAGACTTGGGATCAAATCC |  |
| **Bd 5627 F**  **Bd 6439 R** | GACTTCCAATCATAAGGTCTAC | 812 |
|  | GCTTAAAATAGAGCATAACACTG |  |
| **Bd 6220 F**  **Bd 7014 R** | CTCCAATTAAGGAAGTATGACG | 795 |
|  | GACTGTCTGTTATTCTTTTCGG |  |
| **Bd 6906 F**  **Bd 7746 R** | TATACCTCGCAATATAACTCAACC | 841 |
|  | TAATCGGATTGGGGATGTGG |  |
| **Bd 7554 F**  **Bd 8350 R** | TATAGCAGCAGGTAATCAAGAAG | 797 |
|  | TTAGAGGGGGTAAGATTCGTG |  |
| **Bd 8198 F**  **Bd 8978 R** | AACCTCATTTCATTGACACCAC | 781 |
|  | CCTAAGGCTCATGTTGAAGC |  |
| **Bd 8774 F**  **Bd 9681 R** | AAGCCTTTAAATCAGTTTGACGC | 908 |
|  | GAGTATGTGAAGGTGCTTTGG |  |
| **Bd 9533 F**  **Bd 10317 R** | CACCATTCATAAAACAAATAGGAC | 785 |
|  | AATGCGGCAATTAGTGTAGTAG |  |
| **Bd 10073 F**  **Bd 10859 R** | ATAAATCACCCCTTAGCAATAGG | 787 |
|  | GATCCGTAGTAGATCCCACG |  |
| **Bd 10711 F**  **Bd 11431 R** | GCAGACATCAACTTAGCATTC | 721 |
|  | AGAGGACTAGGGCAATTACC |  |
| **Bd 11204 F**  **Bd 12077 R** | ACCCCTACTTCTCATACAAGG | 874 |
|  | GTGAATCGGAGTTAGTTTCTGG |  |
| **Bd 11861 F**  **Bd 12620 R** | TAACGAAAACGAGGTAAAGTCCC | 760 |
|  | TTTTGGAACGGAAGGTTCTAGG |  |
| **Bd 12529 F**  **Bd 13213 R** | CGCATCACAAAAAGGTTGAGG | 685 |
|  | AGACGAGAAGACCCTATAAATC |  |
| **Bd 13004 F**  **Bd 13929 R** | TCTCCAAAAAAATTACGCTGTTATCCC | 926 |
|  | ATTGTACCTTGTGTATCAGGGTTTATC |  |
| **Bd 13621 F**  **Bd 14518 R** | GCTAATTCTAAGCATACATTTTATATTACC | 898 |
|  | CTGTAATTGATAATCCACGATGAACC |  |
| **Bd 14226 F**  **Bd 14801 R** | ACCTTAATAGCAAGAGCGACG | 576 |
|  | CTAAATTTGTGCCAGCAGCC |  |
| **Bd 14605 F**  **Bd 15135 R** | CATTTTAAATAATAGGGTATCTAATCCTAG | 531 |
|  | TTAATGAAAAACGGTATATTACTTAGGG |  |
| **Bd 14894 F**  **Bd 15850 R** | AGCAAAAATACACGCAAAAACTTAC | 957 |
|  | TTTATACTTACATTTAAGTGGTGTATG |  |
| **Bd 15706 F**  **Bd 472 R** | AATTCTCGGAAATGTCTATATTGG | 682 |
|  | CTGCTGAGGCTATGGCTTG |  |

**Supplementary Table S3.** List of the GenBank accession numbers of the *COI* and *ND4* partial sequences from *B. dorsalis* and *B. carambolae* used in the present study.

| Sample id* | GenBank Accession number | |
| --- | --- | --- |
|  | ***COI*** | ***ND4*** |
| Bd 189 | KC446059 | KC446176 |
| Bd 200 | KC446064 | KC446181 |
| Bd 209 | KC446069 | KC446186 |
| Bd 224 | KC446074 | KC446191 |
| Bd 228 | KC446078 | KC446195 |
| Bd 400 | JX099639 | KC446216 |
| Bd 406 | JX099643 | KC446219 |
| Bd 411 | KC446099 | KC446220 |
| Bd 412 | KC446100 | KC446221 |
| Bd 416 | KC446103 | KC446224 |
| Bd 419 | KC446104 | KC446225 |
| Bd 580 | JX099650 | KC446226 |
| Bd 594 | JX099664 | KC446234 |
| Bd 744 | JX099708 | KC446239 |
| Bd 757 | JX099720 | KC446252 |
| Bd 771 | JX099726 | KC446258 |
| Bd 775 | JX099730 | KC446262 |
| Bd 798 | KC446122 | KC446281 |
| Bd 813 | JX099620 | KC446287 |
| Bd 1080 | KC446127 | KC446301 |
| Bd 1111 | KC446146 | KC446317 |
| Bd 1112 | JX099692 | KC446318 |
| Bd 1115 | KC446147 | KC446321 |
| Bd 1121 | KC446149 | KC446325 |
| Bd 1123 | JX099700 | KC446327 |
| Bd 1126 | KC446150 | KC446330 |
| Bd 1140 | JX099585 | KC446338 |
| Bd 1147 | JX099592 | KC446345 |
| Bd 1170 | JX099604 | KC446356 |
| Bd 1181 | JX099615 | KC446359 |
| Bd 1201 | JX099676 | KC446368 |
| Bd 1209 | JX099684 | KC446376 |
| Bd 1225 | JX099737 | KC446384 |
| Bd 1228 | JX099740 | KC446387 |
| Bd 1234 | JX099746 | KC446393 |
| Bd 1236 | JX099748 | KC446395 |
| Bd 1238 | JX099750 | KC446397 |
|  |  |  |

*from Boykin et al. [24]

**Supplementary Figure S1**

**
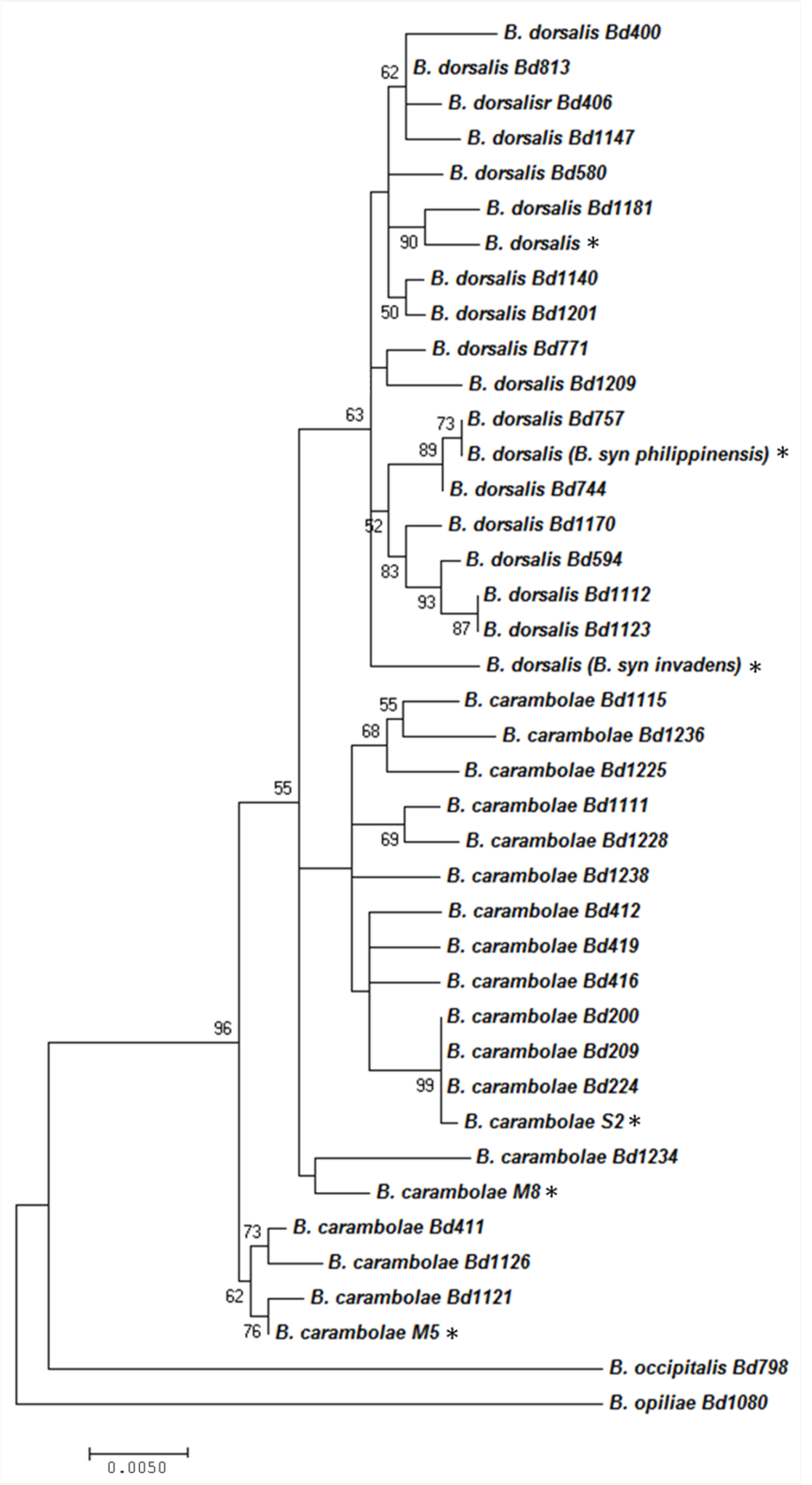
**

**Supplementary Figure S1.** Molecular Phylogenetic analysis by Maximum Likelihood method. Tree based on concatenated partial sequences of the *COI* and *ND4* genes of 38 *B. carambolae* and *B. dorsalis* specimens*.* *Bactrocera opiliae* and *Bactrocera occipitalis* were used as outgroups. The evolutionary history was inferred by using the Maximum Likelihood method based on the Hasegawa-Kishino-Yano model. Codon positions included were 1^st^+2^nd^+3^rd^. The percentage of trees in which the associated taxa clustered together is shown next to the branches; only the ones higher than 50 are presented. The tree is drawn to scale, with branch lengths measured in the number of substitutions per site (Scale bar = 0.005 substitutions per site). Asterisks indicate the sequences analyzed in the present study. Sequences’ accession numbers in Supplementary Tables S1 and S3.
